# Access to Care for Mental Health Problems in Afghanistan: A National Challenge

**DOI:** 10.34172/ijhpm.2021.46

**Published:** 2021-05-24

**Authors:** Viviane Kovess-Masfety, Elie Karam, Katherine Keyes, Ajmal Sabawoon, Bashir Ahmad Sarwari

**Affiliations:** ^1^Department of Psychiatry, McGill University, Montreal, QC, Canada.; ^2^Conseil Santé, Clichy, France.; ^3^Laboratoire de Psychopathologie et Processus de Santé (LPPS), Université de Paris, Boulogne-Billancourt, France.; ^4^Department of Psychiatry & Clinical Psychology, St. George Hospital University Medical, Beirut, Lebanon.; ^5^Faculty of Medicine, University of Balamand Medical Center, University of Balamand, Beirut, Lebanon.; ^6^Institute for Development, Research, Advocacy & Applied Care (IDRAAC), Beirut, Lebanon.; ^7^Mailman School of Public Health, Columbia University, New York City, NY, USA.; ^8^Governance Institute of Afghanistan (GI-A), Kabul, Afghanistan.; ^9^Department of Mental Health & Substance Abuse, Primary Health Care Directorate, Ministry of Public Health, Kabul, Afghanistan.

**Keywords:** Public Policy, Service Research, Epidemiology, Mental Health, Psychiatry, Afghanistan

## Abstract

**Background:** This paper describes the access to care for mental health problems in Afghanistan, according to the nature of the mental health problems and the service provider. Following the Andersen model, it evaluates the respective roles in access to care of "predisposing," "needs," "enabling" factors, and other "environmental" factors such as exposure to traumatic events and level of danger of the place of residence.

**Methods:** Trans-sectional probability survey in general population by multistage sampling in 16 provinces, nationally representative: N=4445 (15 years or older), participation rate of 81%. Face to face interviews using standardized measures of mental health (CIDI, Composite International Diagnostic Interview). Different logistic regression models are presented.

**Results:** The 12-month rate of mental health help-seeking was 6.56% with substantial regional variation (2.35% to 12.65%). Providers were mainly from the health sector; the non-health sector (religious and healers) was also prevalent. Most consultations were held in private clinics (non-governmental organisation, NGO). The severity of mental health disorders as well as the perceived impairment due to mental health were independently very important: odds ratio (OR) = 6.04 for severe disorder, OR=3.79 for perceived impairment. Living in a dangerous area decreased access to care: for high level of danger and for very high level: OR=0.22. Gender, education and ethnicity were not associated with mental health help-seeking after controlling for exposure to trauma.

**Conclusion:** Access to care for mental health problems depended mainly on the needs as defined as disorder severity level and impairment, and on environmental factors such as exposure to traumatic events. The system seems equitable; however, this is counterbalanced by a very challenging environment. This survey is a testimony to the hardship experienced by the Afghan population and by health professionals, and to the efforts to deliver organized mental healthcare in a challenging situation. This research may inform and support policy-makers and NGOs in other countries undergoing similar challenges.

## Background

**Key Messages**

**Implications for policy makers**

- There is no health without mental health.
- Mental health should be integrated into basic services.
- A mental healthcare policy can be instated and is feasible, including in a troubled country such as Afghanistan.
- World Health Organization (WHO) instruments such as the Mental Health Gap manual could provide support for basic health workers without prior mental health knowledge and the use if such support should be encouraged.
- A referral system to a mental health problem should back up primary care in a remote manner whenever necessary.

**Implications for the public**

Mental health problems are very frequent and highly comorbid with physical health problems. In addition, mental health problems are highly discriminated against and stigmatized everywhere but even more so in developing countries. Most importantly, the public is often unaware or ignores that mental health problems are treatable. Mental health problems are often unattended by health policies, especially in developing and war-torn countries. This paper documents how, in one of the most troubled countries in the world, Afghanistan a national mental health policy has been implemented by training primary healthcare workers to address most mental health problems providing them with a backup professional network for the more difficult cases. These elements may contribute to offering the public greater access to care.

Mental disorders are important contributors to the global burden of disease.^1,2^ They cause personal distress and carry major consequences in the functioning of daily life. Low- and middle-income countries are the least able to bear the burden of population morbidity in general, including mental disorders, due to factors including funding, governmental resources, and awareness.^3^ In Afghanistan, years of armed conflicts rendered health systems disorganized^4^ and disproportionally affected the mental health system.^5^

 Despite sustained efforts, Afghanistan, similar to many developing countries, has few mental health specialized resources. The 2017 version of World Health Organization (WHO) Atlas for Afghanistan,^6^ which acknowledged a mental health stand-alone policy (2016) outlined specific indicators to be monitored, including a child and adolescent component. There are few psychiatrists in the region, with an estimated 0.23 per 100 000 and few mental health nurses (0.10 per 100 000) and psychologists (0.30 per 100 000). Only one mental hospital and four psychiatry units in general hospitals were reported in addition to mental health and community based out-patient facilities. An important part of the mental healthcare system belongs to the private sector: many non-governmental organisations (NGOs) operate in Afghanistan. under the supervision of the Ministry of Health, but this renders the system fragile and hard to control in a vast and diverse country.

 Years of conflict have shattered the Afghan mental healthcare situation: after the fall of the Taliban in 2001, there were severe shortages of healthcare staff, supplies, and infrastructure, and the organization of the healthcare system itself was largely insufficient.^7^ The rebuilding of the Afghan healthcare system had to be done from scratch and provided opportunities to integrate mental health into basic health services through the use of funds that became available during the complex humanitarian emergency. Practice-oriented mental health training for general health workers and ongoing clinical supervision in the basic healthcare system led to substantially increased demand for, and access to, basic mental healthcare services, among them counselling for psychosocial problems.

 Afghan Mental health has been one of the Ministry of Public Health’s priorities, and became a part of the Basic Package of Health Service in 2003.^8^ A national mental health strategy was developed for five years (2010-2014)^9^ and revised in 2015. The strong point of this strategy was the integration of mental health services into each level of the healthcare system. among them the Basic Package of Health Service, which described minimum interventions to be provided at various levels of the general healthcare system. Further introductions were, at a second level, the Essential Package of Hospital Services, followed by specialty hospitals at tertiary level. The system promotes a strong referral system, for mental health problems, at the three tiers of the healthcare system. The lowest level is the “health post” where “community health workers” (CHWs) have basic mental healthcare training. Then, at a higher level, the basic health centers which integrate mobile health team, are small health facilities staffed by nurses, community health supervisor and, for some of them, by a doctor, covering a population of 15 000 to 30 000 people. This followed by comprehensive health centers, for 30 000 to 60 000 people, staffed by physicians, nurses and midwives who were trained to identify the most important psychiatric disorders and to formulate a treatment plan. In the developed strategy, physicians received additional training in the appropriate prescription of psychotropic medication and nurses and midwives received additional training in basic psychosocial interventions, such as psychoeducation for patients and family members. Outpatient and inpatient services were made accessible in district hospitals.

 As a theoretical framework to examine service utilization, the Andersen behavioral model of health service treatment contact is the most extensively studied model thereof service utilization. This model posits that service utilization is a function of (*a*) “predisposing” characteristics, (*b*) “enabling” factors and (*c*) “need” for services.^10^ However, in a recent review, these various factors have been further elaborated upon.^11^ Most of the research in this area has defined “predisposing factors” as socio-demographic characteristics: gender, age, marital status, ethnicity and education; “enabling factors” as factors which facilitate individual service use such as income, availability and accessibility (for example, time to reach a health post); “need factors” as the presence of a disorder, its severity level and the perceived need for help which may motivate service use. The model has been adapted to developing countries by adding external “environmental factors”^12^ such as place of residence (rural/urban region) that potentially modulate use of care.

 Contrary to many studies using secondary data that decreases the potential number of variables in each dimension of the Andersen model,^11^ the present report is based on data from a unique, extensive national survey, measuring the most common mental health disorders and access to care that was rendered possible by a European Union grant in the context of recent national Afghan mental health strategy. More specifically, the present study investigates how equitable access to care actually is, through an examination of the relative importance of key predisposing, enabling and needs factors.^10^ In the context of Afghanistan, we propose to add “environmental factors” (place of residence: rural/urban, region, level of danger and personal exposure to potentially traumatic events). We make the assumption that equitable access should depend mainly on needs, defined as the presence of mental health problems, their level of severity and the perception of impairment created by the mental health problems, despite the context, whereas an inequitable access will be determined by education, ethnicity, region and level of danger.

## Methods

###  Design

 The household survey was implemented in each of the 8 regions of Afghanistan: (1) Eastern, (2) South Eastern, (3) Southern, (4) Western, (5) North Western, (6) North Eastern, (7) Central Kabul, and (8) Central Bamiyan. A multi-stage stratified cluster sampling method was applied: in each region two provinces were randomly selected, totaling 16 provinces out of the 34 provinces of the country; then the Central Statistical Organization, after random sampling of clusters for each selected province, provided the list and maps of 320 randomized clusters. However, considering ongoing safety issues, approximately 10 clusters had to be replaced with a nearby cluster in the same district or the next district. The surveyors were then required to complete questionnaires for 14 households randomly selected in each cluster. In the household, a randomized adult selection was based on Kish selection. The sample interviewed involved Afghan males and females at least 15 years old and residing in the selected cluster.

###  Sample

 Based on an estimated prevalence of 20% of mental health problems, anticipating a 10% refusal rate and taking in account the design, the sample size needed by region was 542, which resulted in a minimum total of 4336 persons for the whole country (with the true estimates to be captured within a 95% confidence interval). The total number of participants who completed the survey was 4445. Participation rate was on average 90% for households (86 to 93% depending on the region) and 81% for individuals (50 to 100% depending on the region).

###  Population

 The Afghan population is a young population: 48% of the sample was below 35 years old and only 4.47% were 65 years or older. However, the sample differs slightly from the total Afghan population as described by the recent official estimate (Estimated Population of Afghanistan 2107-2018, Central Statistics Organization Islamic, Republic of Afghanistan, 2017) survey, so a weight was established to address discrepancies related to age and gender.

 The share of urban areas was around 10% to 50% among selected clusters, except Kabul Province, where urban areas represented around 70% of the clusters including Kabul city. Overall, 73.10% of the sample lived in rural areas, a rate comparable to the total Afghan population (70.9% in rural, 20% in urban; the remaining 6% being nomads). In addition, 59% of the population had no income; 7.67% had a monthly income over 10 000 AFN (200$). On the total weighted sample: 27.55% were Tajik, 47.82% Pashtu, 11.4% Hazara, 6.58% Uzbek, and 6.65% of another ethnicity (for 0.08% the information was missing). As expected, ethnicities were very different across regions. Lastly, 52.62% of the weighted individual sample completed no formal education and did not have any reading skills, 3.5% did not complete primary school, 6.4% completed primary, 8.47% secondary school, 18.1% some college and 7.85% university.

###  Instruments

 The collected socio-demographic information included: gender, age, marital status, educational level, occupation (position and sector), income and ethnicity.

 For Major depressive episode and generalized anxiety the Composite International Diagnostic Interview (CIDI) Short Form developed by Kessler et al^22^ was administered, supplemented by questions on impairment (Sheehan scales on four domains quoted from 0 to 10) plus questions on mania and on suicidal behaviors, and the CIDI psychosis module, which included questions regarding 6 psychotic experiences: 2 related to hallucinatory experiences (visual and auditory) and 4 related to delusional experiences.

 CIDI and Sheehan translations to Dari, equivalent to the Iranian language, were available,^23^ whereas we had to translate the instruments into Pashtu. This translation was focused on cross-cultural and conceptual, rather than on linguistic/literal equivalence by using forward-translations and back-translations as recommended by WHO (see http://www.who.int/substance_abuse/research_tools/translation/en/, http://www.who.int/substance_abuse/activities/assist/en/). A validation procedure was completed on a clinical population in both languages comparing instrument results with psychiatrist’s evaluations using a DSM-5 (Diagnostic and Statistical Manual of Mental Disorders, fifth edition) checklist with satisfactory results.

 Psychological distress and impairment due to mental health problems were measured by two 36-Item Short Form Survey (SF-36) subscales: MH5 (Psychological Distress) and RE (Role Emotional), which is perceived impairment due to mental health, for which we applied the recommended threshold.^13-15^ The Life Event Checklist five^16^ was used to assess lifetime exposure to 16 events known to potentially result in post-traumatic stress disorder (PTSD) or distress, as well as the PTSD Checklist For DSM-5, a 20-item self-report measure that assesses the 20 DSM-5 symptoms of PTSD.^17-19^

 In addition we built a “number of traumatic events” variable: none, one to three, four or more traumatic events either self-experienced or witnessed, in accordance with the fact that cumulative exposure is critical in the risk of PTSD.^20^

 The Alcohol, Smoking and Substance Involvement Screening Test (ASSIST)^21^ (see https://www.who.int/substance_abuse/activities/assist/en/) from the WHO^21^ was used to detect substance use and related problems. Two indicators were built following ASSIST quotation: mild substance problem that requires brief intervention for any product except tobacco, and severe problem which requires more intensive treatment. A translation in Dari was available from WHO and a Pashtu version was available from a previous study.

 The module on help-seeking for mental health problems followed. It was introduced by: “Did you seek help for any mental health problem we talked about during the interview?”; a list of the mentioned problems was proposed, followed by a question on the type of provider either from the health system, such as physician, psychosocial counsellor or CHW or outside the health sector (non-health) such as Imam or healer; the location where the contact with the provider happened. and the level of satisfaction (scored from 1 to 5 very satisfied). Questions regarding hospitalization included duration, place and satisfaction.

###  Procedure

 Interviews were administered either in Dari or Pashtu according to the language spoken in the selected household. Language/ethnic specificities in the different provinces were taken into consideration by recruiting interviewers fluent in either language. Questionnaires were read aloud to participants.

###  Data Analysis 

 Algorithms were applied to the CIDI to extract DSM 5 diagnostic criteria as in the World Mental Health (WMH) study^24^. Similarly, a severity indicator was built on the WMH surveys model^25^: (1) severe was defined as either presence of mania, a suicidal attempt plus any 12-month disorder, any substance dependence, more than one disorder and a high level of impairment of Sheehan scales, (2) moderate was defined as more than one disorder and moderate level of impairment or substance disorder without dependence, and (3) low severity was defined as any low severity 12-month disorder.^25^

 A summary variable, “Any mental health problem,” was also built which was considered positive in the presence of either any 12-month diagnosis, any mild or severe misuse of substances, any 12-month psychotic experience or suicidal thoughts or any declared impairment due to any mental health problem (from RE SF-36 subscale).

 Analyses were performed with STATA 15.1. Since the composition of the sample obtained differed from the Afghan population, tables are presented weighted on gender and age compositions. Risk factors are presented in univariate and multivariate logistic regressions.

## Results

###  Description of Access to Care and Care Providers by Mental Health Problem

 Among those who ever sought help, 1.18% declared either “self-help,” “somebody who was a family member or a friend,” or “other.” If the latter was the only type of help sought, it was considered that help was not sought.

 Overall, 10.74% (CI: 9.83-11.72) of the population received help for their mental health problems over the course of their life (lifetime), with important regional differences: ranging from 4.5% in the Central highland region to 21.82% in the South. In the past year, 6.56% received help (CI: 5.83-7.74%) again with large interregional differences, ranging from 3.1% in central highland to 11.3% in the South.

 50.46% of the population reported some 12-month mental health problems as defined “any mental health problem”, (CI: 48.88%-52.04%) with large interregional differences: 36.26% in the North to 67.83% in the West.

 Among those reporting any mental health problem, 18.52% received help at some point, 12.28% in the past 12-months.

 Receiving help correlated with 12 month and lifetime diagnoses. In addition, suicidal thoughts during the past month were the highest predictor, followed by having a major depressive episode, mania or severe addiction. Psychotic experiences had the lowest rate for help-seeking.

 Help providers were mainly from the health sector but the non-health sector (religious leader or healer) was also important. Health and non-health help were mutually exclusive among half of the help-seekers. Non-health providers were frequently accessed for severe addiction: 30.32% went to the health sector and 18.79% to the non-health sector, 10.59% exclusively to this latter. Persons with suicidality also sought help from the non-health sector: 12.36% for 12-month suicidal thoughts, 4.86% exclusively. The proportions of non-health providers were lower for the other types of disorders but remained around 9% for most of them (Table 1).

**Table 1:** Help Received by Types by Diagnosis (N = 4384)

	**Prevalence %** **(Number) **	**Lifetime **					**12 Months**
		**Any Help%**	**Any Health Provider %**	**Any Non-health** **Provider %**	**Non-health Provider Only %**	**Hospitalization % **	**Any Help 12 mon**
MDE 12 mon	11.71 (545)	32.60	29.05	8.50	3.55	4.80	25.23
Mania^a^ LT	0.83 (33)	39.76	35.60	8.52	4.16	7.48	20.38
PTSD 12 mon	5.34 (270)	27.74	24.33	8.99	3.41	4.35	17.43
GAD 12 mon	3.38 (161)	34.31	29.99	9.45	4.32	4.75	21.51
At risk substance use mon	8.22 (409)	30.76	26.23	9.40	4.53	5.42	20.05
Addiction 3 mon	2.18 (105)	40.91	30.32	18.79	10.59	11.64	22.56
Psychotic experience 12 mon	27.57 (1122)	19.86	17.43	4.57	2.43	3.24	12.80
Suicidal thoughts 12 mon	2.26 (105)	41.92	37.06	12.36	4.86	12.71	22.27
Suicide attempt life time	3.42 (159)	36.39	31.42	11.79	4.97	9.65	21.86
Impaired by mental health	39.44 (1848)	18.99	17.11	3.82	1.87	2.56	12.27
No severity	86.25	6.94	6.37	0.89	0.06	0.07	3.75
Mild	3.03	23.44	20.41	4.32	3.03	2.59	17.35
Moderate	6.02	25.79	23.17	5.66	2.62	3.25	18.91
Severe	4.69	40.31	33.28	15.57	7.03	10.34	29.20

Abbreviations: MDE, major depressive episode; GAD, generalized anxiety disorder; PTSD, post-traumatic stress disorder. ^a^Mania N = 3674 due to missing values; weighted % (raw numbers).

 Hospitalization trends followed the help-seeking behavior: the highest was predicted by 12-month suicidal thoughts and severe addiction.

 5.44% of the sample reported lifetime contact for any mental health problem with a physician, 4.22% with a CHW, and 1.75% with an Imam; psychologists or psychosocial counsellors were quite rare: 0.37%, less frequent than healers: 0.69%.

 These rates were higher for those who had any mental health problem: 10.01% received help from a physician, 6.68% for the CHW, 3.28% for the Imam, 1.39% from a healer. Health sector professionals were more frequently accessed as compared to non-health professionals: 16.48% versus 3.87%, among them 2.03% were exclusively non-health.

 The severity of the disorders predicted both types of care: healthcare as well as non-health. Satisfaction (from 1 to 5) was moderate to high: 3.95 to 3.52 with no significant differences across provider types (Table 2).

**Table 2:** Help Received by Type of Provider

	**Total Population N = 4384%**	**Among AMHP + N = 2293%**	**Among Helped AMHP + N = 442%**	**Total Population Receiving Help (N = 438)**				**Satisfaction** ^a^	**95% CI**
				**Severe**	**Moderate**	**Mild**	**None**		
Psychologist	0.37	0.70	3.76	1.88	0.93	0.36	0.19	3.59	2.85-4.33
CHW	4.22	6.68	35.93	11.55	6.06	5.70	3.30	3.90	3.75-4.06
Doctor	5.44	10.01	53.85	23.46	15.23	12.41	2.48	3.79	3.64-3.93
Religious	1.75	3.28	16.59	13.44	3.20	2.74	0.67	3.67	3.44-3.90
Healer	0.69	1.39	7.50	5.03	2.20	1.15	0.16	3.52	3.15-3.89
Any help (life time)	10.67	18.52		40.06	22.97	20.31	6.42	3.85	3.75-3.95
Any help (12 mon)	6.48	12.28		29.23	16.67	14.34	3.40	3.95	3.82-4.08
Any health professional	9.55	16.49	88.70	34.11	20.62	18.20	5.87	3.85	3.74-3.96
Any non-health provider	2.03	3.84	20.66	14.18	4.57	3.16	0.77	3.65	3.43-3.87
Any no heath prof exclusive	1.11	2.03	10.89	5.95	2.35	2.11	0.55	3.80	3.54-4.07
Any Lay help	1.72	3.10	9.57	9.93	3.64	1.87	0.89	3.87	3.63-4.10

Abbreviations: AMHP, any mental health problem; CHW, community health worker. *Note*. Health = CHW, psychiatrist, psychologist or counsellor; Non-health = Imam or Healer. ^a^ From 5 very satisfied to 1 very dissatisfied.

 Most of the consultations were held in private clinics: 50.32%, followed by 27.26% in government clinics, 8.67% at a hospital, 7.4% at the place of the provider and 6.35% in other places, which were mainly in the private home of one of the patients or a family member, or a friend.

 1.34% of the total sample and 11.95% of those who received help were hospitalized at least once for a mental health problem; 45.4% of those hospitalized were in general governmental hospitals, 43.27% in private hospitals or clinics, and 11.33% in the psychiatric government hospital.

###  Role of Predisposing Factors 

 In univariate analyses, gender was not linked to seeking help. Age was a strong predictor: with those aged 15-34 years displayed the lowest rate at 5.9%, those aged 35-59 years the highest at 9.21%, and those 50 and over the middle at 7.93% (*P* = .0012). Educational level was a predictor (*P* = .039): the non-educated had the highest rate at 7.44%. Ethnicity was important: Pashtun had the highest help seeking rate, at 8.83%, followed by those who did not belong to the most common ethnicities (7.64%), and the Tajik, at 5.57%. Uzbek and Hazara ethnicities had the lowest rate at 3.50% and 4.30% (*P* = .0002).

 Multivariate analyses partially reflected these trends: age and ethnicity became the only predictors; the middle aged had a greater probability as compared to the young OR = 1.50, and Pashtun had an odds ratio [OR] = 1 compared to Tajik; educational level was no longer significant, since it is very much linked to ethnicities; 63.95% of the Pashtun and 61.05% of the Uzbek have no education whereas this percentage was 42.05% for the Uzbek and 50.3% for the Tajik (Table 3).

**Table 3:** Model 1: Last 12-Month Care “Predisposing” Determinants

**Last 12-Month Access to Care**	**OR**	**Adjusted OR**	**95% CI **		***P*** ** Value**
			**Lower **	**Upper**	
Gender, female/male	1.02	0.97	0.74	1.27	.834
Education					
No school or less than primary					
Primary or secondary	0.60^a^	0.73	0.46	1.16	.188
High or University	0.78	0.92	0.64	1.32	.654
Age					
15-34					
35-49	1.62^a^	1.50^a^	1.12^a^	2.02^a^	.007^a^
≥50	1.27	1.21	0.85	1.70	.290
Ethnicity					
Tajik					
Pashtun	1.57^a^	1.51^a^	1.12^a^	2.04^a^	.007^a^
Hazara	0.54	0.56	0.30	1.04	.066
Uzbek	0.62	0.60	0.29	1.23	.164
Other	1.35	1.32	0.76	2.28	.328

Abbreviation: OR, odds ratio. ^a^ *P* ≥ .05.

###  Role of the “Need Factors” Model II

 Once the clinical predictors were added to the socio-demographic predictors, their influence was not modified, but clinical variables had a much higher effect: the severity of the disorder had a major role as did perceived impairment (RE) due to mental health problem (Table 4).

**Table 4:** Predisposing and Need Factors Model II

**12 Months Access to Care**	**OR**	**Adjusted OR**	***P*** **>** ***t***	**Adjusted OR [95% CI]**	
Gender					
Female/Male	1.02	0.87	.366	0.65	1.17
Education					
No school or less than primary					
Primary or secondary	0.60^a^	0.73	.238	0.44	1.23
High or University	0.78	1.06	.786	0.71	1.56
Age					
15-34					
35-49	1.62^a^	1.43^a^	.035^a^	1.03	2.00
≥50	1.27	0.99	.949	0.67	1.46
Ethnicity					
Tajik					
Pashtun	1.57	1.55	.011^a^	1.10	2.17
Hazara	0.54	0.67	.237	0.34	1.30
Uzbek	0.62	0.77	.543	0.34	1.77
Other	1.35	1.02	.955	0.53	1.95
Severity					
None					
Mild	5.45^a^	3.19	.000^a^	1.79	5.68
Moderate	5.93^a^	4.87	.000^a^	3.19	7.44
Severe	10.19^a^	5.48	.000^a^	3.65	8.22
Perceived impairment	5.90	4.23	.000^a^	3.02	5.91

Abbreviation: OR, odds ratio. ^a^ *P* ≥ .05.

###  Role of Enabling and Environmental Factors Models III and IV

 The “enabling factors,” namely income level and distance to a basic health post, were not significant predictors. In contrast, “environmental factors” such as the level of danger of the areas and the region were very important and annulled the role of ethnicity, without decreasing the role of the “need factors” including the severity of the disorder and perceived impairment. In addition, exposure to four traumatic events or more increased access to care (OR = 4.24). Residing in the West province independently decreased receiving care as compared to the Central Kabul region (Table 5).

**Table 5:** Predisposing, Need, Enabling and Environmental Factors Models III and IV

	**12 Months Access to Care **	**OR**	**Adjusted OR**	***P*** **>** ***t***	**Adjusted OR (95% CI)**	
Predisposing factors	Gender F/M	1.02	0.98	.913	0.63	1.52
	Education					
	No school or less than primary					
	Primary or secondary	**0.60**	0.64	.107	0.37	1.10
	High or University	0.78	0.87	.536	0.56	1.36
	Age					
	15-34					
	35-49	**1.62**	1.35	.113	0.93	1.95
	≥50	1.27	1.04	.874	0.67	1.59
	Ethnicity					
	Tajik					
	Pashtun	**1.57**	1.06	.838	0.63	1.76
	Hazara	0.54	0.91	.840	0.38	2.20
	Uzbek	0.62	1.19	.685	0.51	2.78
	Other	1.35	1.16	.699	0.55	2.42
Need factors	**Severity**					
	**None**					
	**Mild**	**5.45**	**3.81**	.000	2.08	6.97
	**Moderate**	**5.93**	**6.41**	.000	4.02	10.24
	**Severe**	**10.19**	**6.04 **	.000	3.81	9.57
	**Perceived impairment **	**5.90**	**3.79**	.000	2.68	5.37
Enabling	Income					
	No income					
	≥3000	1.15	1.10	.750	0.60	2.02
	3001-6000	1.10	0.91	.734	0.51	1.61
	6001-10 000	1.44	1.25	.395	0.75	2.10
	>10 000	1.00	0.69	.305	0.35	1.39
	Time to get to health post: <0.5					
	1 hour	0.96	0.99	.942	0.67	1.45
	>1 hour	0.92	0.79	.327	0.50	1.26
Environmental factors	Rural/Urban	1.01	0.84	.398	0.57	1.25
	Region					
	Central/Kabul					
	South	**2.09**	0.84	.775	0.26	2.75
	East	0.98	0.96	.916	0.49	1.91
	South West	**1.59**	2.06	.244	0.61	6.91
	West	1.10	**0.23**	.019	0.07	0.78
	North	0.79	0.32	.068	0.10	1.08
	Central High Land	**0.34**	0.32	.096	0.09	1.22
	North East	**0.47**	0.62	.214	0.30	1.31
	Danger					
	Low/middle					
	High	**0.64**	**0.22**	.000	0.12	0.40
	Very High	**0.56**	**0.22**	.012	0.07	0.72
	Exposure none					
	1 to 4 events	**2.43**	1.84	.073	0.94	3.58
	≥4 events	**4.23**	**2.45**	.011	1.23	4.87

Abbreviation: OR, odds ratio. Bold significant, *P* <.05

## Discussion

 The 6.56% 12-month rate of mental healthcare access, formal and unformal, is similar to what was found in the WMH Middle-income group of countries: Columbia 5.5%, Mexico 5.1%, Lebanon 4.4%, as well as in some high-income countries: most European countries reported from 4.3% to 11.3%; the United States being by far the highest at 17.9%. The rate is much higher than in a low-income country such as Nigeria: 1.6%.^25^ Importantly, considerable variation was found from one Afghan region to another ranging from 2.35% in central Highland up to 12.65% in the South.

 Overall access to services comprised people with different levels of disorder severity, while most of the available resources should be directed towards those with the greatest needs. Respectively, 29.23% of the most severe, 16.67% of moderate cases, 14.34% of mild cases and 3.40% of those with no identified mental health problem reported, received help. For severe disorders, this is by far lower than in Western countries, but remains higher or similar to low middle and high middle countries such as Lebanon, where the rates are 20.1% for severe cases and 11.6% for moderate, or some countries such as Mexico or Columbia, or South Africa with 26.2% for severe cases.^25^ The participation of religious providers was 3.28% in the Afghan sample versus for example 4.7% in the Lebanese sample, whereas healers had lower rates in Afghanistan: 0.14% vs 0.6% in Lebanon. As a whole, among providers outside the health system, exclusive health seeking to both religious providers or healers was relatively low in these countries: the religious providers 2.04% in Afghanistan versus 1.6% in Lebanon.

 Following the Andersen model, we did not find gender, education or income as significant predictors of access to care for mental health problems, contrary to what was found in the Lebanese study.^26^ Severity of disorder and self-reported impairment were the main predictors of access, which is coherent with a more recent Lebanese study.^27^ However, in contrast with the latter study, income had no influence on help-seeking. In Afghanistan, the main determinants of access to care for mental health problems were the clinical needs: level of severity and need perception. It appears as though predisposing factors and facilitating factors did not to play a significant role. This could be interpreted as equalitarian access where neither age, education, income nor ethnicity play a role.

 Once using the model adapted to developing countries,^12^ environmental factors such as the level of danger, exposure to traumatic events and region play a major role, in addition to clinical needs. The important role of environment, although expected, seems to create inequalities across regions. This is a true challenge for the Afghan ministry of health, despite its noticeable efforts deployed to render mental healthcare accessible, and adds to the burden and risks of the people living there.

 Importantly, in addition to factors which preclude access to care, there are risk factors for mental health problems. Specifically, in this survey, the main risk factors were to be female, aged 35 years or older, exposed to traumatic events and living in an area with a high level of danger,^28^ but we did not find gender as an independent factor for access to care. Women are more likely to suffer from mental health problems, though at the same level of suffering, women seem to have equal access; therefore, it is necessary to work on preventive measures to protect women from mental health problems. However, the risk factors could intertwine in a more complex manner; exposure to trauma is a risk factor for mental health problems as well as for access to care. As a result, those who are at risk because of exposure seem to have higher needs and access but, as living in highly dangerous areas, this access remained less available than in less dangerous areas, for the same level of exposure. These considerations require attention for healthcare planning, which may need to prioritise those who have the highest needs, as a complex combination of risk factors for mental health problems and for the absence of access to care for these problems.

###  Limitations

 Conducting a survey on a national representative sample in Afghanistan is a challenge for several reasons. First, with extreme level of danger and accessibility comes potential for sampling bias. Although participation response rate was high, some highly dangerous areas of the country were excluded from the study. Second, the classification of danger levels was based per regional number of attacks, that may vary inside the same region and render the level of danger unprecise. Third, the translation of the instruments in the main languages, and their survey administration to a mainly illiterate population, were additional challenges. The WMH surveys had demonstrated the feasibility of such studies in relatively similar countries, though this does not preclude a misunderstanding of some questions, nor does it limit retrospective recall problems regarding exposure to traumatic events or access to care. In addition, diagnoses and evaluation of severity are based on self-report only; despite the use of validated instruments, this also constitutes a limit. Despite these challenges, the survey reached a remarkable response rate and was deployed in very hard-to-reach areas, providing unique valuable information on access to care.

## Conclusion

 Globally, mental health services in Afghanistan appeared relatively accessible, at least at the same rate as in low middle and high middle countries and with no difference concerning sex, education or income, whereas needs for care were the main determinants of help seeking.

 This positive result was hampered by regional variations; in addition, it seemed that the people living in the most dangerous areas had the lowest access to care, despite their exposure to potentially traumatic conflict; efforts should be made to implement services into these areas as a priority.

 This survey testifies to the extreme hardship experienced by the Afghan population and by the health professionals who provide care to the most in need, along with the remarkable efforts deployed to provide some organized mental healthcare in such a challenging situation.

 It will be important to continue this effort and to monitor access to care in the different parts of the country as the political situation evolves. Some information on a representative sample of the healthcare system at its different levels is also needed to monitor the implementation of basic mental health knowledge and care.

## Ethical issues

 The project was approved by the Afghanistan Institutional Review Board. National Public Health Institute, Ministry of Public health on 12/31/2016 (IRB n° 3355421). A consent form was to be read out loud and obtained from each selected person before inclusion in the study; those who did not accept were not included.

## Competing interests

 Authors declare that they have no competing interests.

## Authors’ contributions

 VKM designed the work, conducted the analyses, drafted the work, approved the final version and agreed on accountability of all aspects. EK participated to the interpretation of data for the work, revised the work bringing important intellectual content, approved the final version, and agreed on accountability of all aspects. KK participated to the interpretation of data for the work, revised the work bringing important intellectual content, approved the final version, agreed on accountability of all aspects. AS collected the data, revised the work bringing important intellectual content, approved the final version, and agreed on accountability of all aspects. BAS participated to the interpretation of data for the work, revised the work bringing important intellectual content, approved the final version, and agreed on accountability of all aspects.

## Funding

 Funding was provided by the European Union Grant: EuropeAid/137-728/DH/SER/AF/, Conseil Santé Contract service N°DCI-ASIE2015/371-619.
